# A cost-benefit analysis of WildFireSat, a wildfire monitoring satellite mission for Canada

**DOI:** 10.1371/journal.pone.0302699

**Published:** 2024-05-23

**Authors:** Emily S. Hope, Daniel W. McKenney, Lynn M. Johnston, Joshua M. Johnston

**Affiliations:** Great Lakes Forestry Centre, Canadian Forest Service, Natural Resources Canada, Sault Ste Marie, Ontario, Canada; University of Klagenfurt, AUSTRIA

## Abstract

In anticipation of growing wildfire management challenges, the Canadian government is investing in WildFireSat, an Earth observation satellite mission designed to collect data in support of Canadian wildfire management. Although costs of the mission can be reasonably estimated, the benefits of such an investment are unknown. Here we forecast the possible benefits of WildFireSat via an avoided cost approach. We consider five socio-economic components: suppression costs (fixed and variable), timber losses, property, asset and infrastructure losses, evacuation costs, and smoke related health costs. Using a Monte Carlo analysis, we evaluated a range of possible changes to these components based on expert opinions. The resulting Net Present Value (NPV) estimates depend on the presumed impact of using WildFireSat decision support data products, with pessimistic and conservative assumptions generating mission costs that typically exceed potential benefits by 1.16 to 1.59 times, while more optimistic assumptions generate benefits in excess of costs by 8.72 to 10.48 times. The analysis here excludes some possibly significant market and non-market impacts expected from WildFireSat due to data limitations; accounting for these additional impacts would likely generate positive NPVs under even cautious impact assumptions.

## Introduction

In the future, increased fire weather severity [1] and longer fire seasons [2] are likely to lead to more wildfire activity [3,4], driving an increase in fire-related management and social costs [5,6]. Despite relatively successful suppression efforts in North America [7], adapting to increased fire activity will require wildfire management agencies to adopt innovative and cost-effective approaches to address current and future challenges [5,8,9]. Equipping decision-makers with the best available data in active fire environments could help reduce negative wildfire impacts, lower wildfire management costs, and generally decrease societal losses to wildfires.

Canadian government researchers have proposed ‘WildFireSat’, an Earth observation mission consisting of three polar orbiting satellites with onboard multi spectral imaging sensors specifically designed to remotely measure the data needed for Canadian wildfire management [10]. WildFireSat will join other polar orbiting observational platforms providing data that supports wildfire management [11]. However, it will be the first operational, purpose-built wildfire monitoring satellite mission optimized for wildfire observation in Canada. Existing fire data products (e.g. those based on geostationary and polar orbiting satellite systems not purpose-built for wildfire monitoring with overpass times outside of the daily peak burn periods) have limited utility for operational Canadian wildfire management needs [12–14]. These systems fail to provide the necessary data detail, periodicity, and scope to be of practical use [10].

WildFireSat has been designed to address many of these issues by providing improved temporal resolution observations of Canada during the crucial daily peak burn period, addressing an important coverage gap. The satellite orbit will be designed to provide national fire observations in near real-time (~30 minutes after overpass), twice a day. The first overpass will occur locally during the early morning period, while the second occurs during the late afternoon typical peak-burn period [10]. Data will be collected via a microbolometer-based imaging sensor capable of accurately measuring thermal energy emitted from wildfires. Unlike traditional cooled thermal detectors, microbolometers are uncooled, making them comparatively inexpensive, lighter and less power demanding, and therefore ideal for small satellite applications (see [15] for a more detailed description of microbolometer technology in the context of wildfire management and remote sensing).

The WildFireSat mission is intended to be a comprehensive wildfire monitoring system including space and ground segments. Data collected by the satellites will be converted into decision support data products that provide actionable strategic intelligence and are expected to include reports detailing fire detections and intensity, spread rate and direction, smoke forecasting and mapping, and predictions of the effectiveness of various suppression tactics (see [10] for a detailed list). Insights provided by these products could decrease the uncertainty faced by on-the-ground decision-makers, directly affecting wildfire management and suppression decisions and generating possible efficiency gains [16]. For example, WildFireSat supported decisions could lead to repurposing of aerial wildfire detection patrols for other tasks, earlier evacuation of threatened communities that decreases social and economic costs, or optimization of suppression resource allocation to achieve earlier containment objectives. The satellite data will also provide a national perspective of fire events across the country, better facilitating resource sharing needs between fire management agencies [9].

WildFireSat mission costs will include development, launch, operations and maintenance, data product development and end user engagement; these costs are not trivial and should be considered in conjunction with any anticipated benefits. Although these values can be estimated based on past satellite construction and existing satellite cost forecast models [17], possible benefits within the context of wildfire management are more challenging to quantify. The literature contains multiple approaches to evaluating the benefits of information, including remote sensing data. The most common satellite impact assessment approaches include “value of information” analyses that assume the benefits of novel information are proportional to the size of industry/sector impacted, and have been used in the past to estimate the economic benefits of available Earth observational data in the context of wildfire [16,18–20]. Pre-launch analyses are less common within the broader, non-wildfire related literature [21], and generally rely on more traditional cost-benefit analysis methods and case studies to guide investments in Earth observational systems [22–24]. Bard [25] concluded that the NPV of remotely sensed data could vary between positive and negative outcomes, depending on data reliability and assumed changes in natural resource management (-$146.9 million to $558.6 million, CAD, 2019). Christensen [26] quantified the economic benefit of using drones to surveille wildfires via expert elicitation, and determined that suppression cost savings were possible, depending on assumptions about the use of the resulting data. Regardless of analysis methodology or timing, wildfire-related remote sensing data have been found to generate positive impacts, improve management outcomes, and decrease fire-related costs [27].

Here we compare the anticipated costs of WildFireSat (development, launch and operations) against a particular set of possible economic benefits flowing from the use of WildFireSat decision-support data products within a recent actual wildfire management context. We estimated use and some selected downstream benefits under various assumptions about the impact of the mission on wildfire management decisions, drawing on a range of expert opinions about WildFireSat decision-support data products and allowing for possible positive and negative impacts. Ultimately, we aim to provide a quantitative evaluation of the possible net economic value of the mission. Although WildFireSat decision-support data products are likely to generate benefits in other contexts (e.g., decreased economic losses due to avoided wildfire-related business closures, reduced impacts of wildfires on environmental services and values, avoided social costs associated with wildland fire evacuations or smoke impacts, etc.), quantifying these particular categories are beyond the scope of the current analysis but may be considered in the future.

## Methods and data

The benefits of WildFireSat are estimated as counterfactual avoided costs or losses brought about by use of the satellite decision-support data products and compared to the direct mission costs. Our analysis assumes that the current use of existing Earth Observational data products (see [28] for an analysis of the use of EO data within wildland fire management) will continue but WildFireSat decision-support data products will be used in conjunction with existing products. We created a computer simulation model to evaluate the costs of Canadian wildfires over a five-year period, with and without an operational WildFireSat over the same period. Comparing these cost data with and without WildFireSat allowed us to estimate the avoided costs or losses (benefits) of the satellite mission. We then compared these benefits with the satellite mission costs (inclusive of design, construction, launch and operational incurred over an eleven-year lifespan) to complete the cost-benefit analysis.

The model relies on @Risk, a Monte Carlo simulation tool [29]. This software replaces static model values with dynamic variables drawn from user-defined distributions, producing a range of possible outputs across multiple iterations. This approach captures some of the uncertainty associated with our assumptions about a future satellite investment that involved stochastic outcomes. We iterated the model 50,000 times to illustrate the range of net outcomes possible with this set of assumptions. At this number of iterations, the model achieved convergence such that all output distribution metrics were stable; additional iterations would provide no new information. Building on the Monte Carlo-based approach, we also employed an extensive sensitivity analysis to explore the implications of larger important model variable adjustments. Results are reported as net present values (NPV) that capture the mission costs and benefits of WildFireSat over its entire lifecycle.

Our approach is similar to those used by Bard [25] and Gould et al. [30]. Bard [25] valued Earth observation data by exploring the anticipated impacts of remotely sensed data on natural resource management decision outcomes, comparing with- and without-data scenarios developed from case studies. Similarly, Gould et al. [30] evaluated the economic benefit of improvements in the Canadian Forest Fire Danger Rating System–a foundational fire management decision support tool used across Canada [31]–by comparing estimates of avoided costs and losses brought about by the investment in the decision support tool as estimated from expert elicitation. The basic approach can be summarized via Eq 1:

$$Net\;Economic\;Value=\sum_{t=1}^{11}\left(\frac{\sum_{i=1}^{n}{Avoided\;Losses}_{i,t}}{{\left(1+r\right)}^{t}}-\frac{{Mission\;Costs}_{t}}{{\left(1+r\right)}^{t}}\right)$$ (1)

where *t* is the lifespan of the satellite mission (design to operation, inclusive), *i* is each cost component investigated, *n* is the number of cost components examined, and *r* is the discount rate. Details on the mission costs, benefits, and sensitivity analysis are provided below.

### Mission costs

The Canadian Space Agency is spearheading the satellite segment of the WildFireSat constellation (design, construction, launch and operations), which will be delivered in large part through industrial partnerships. The current timeline allows for a five-year development/construction period, followed by launch and a five-year operational lifespan. The Canadian Space Agency is also involved in the ground segment of the mission, which includes securing the infrastructure necessary to download the data produced by the constellation. Natural Resources Canada (specifically the Canadian Forest Service and the Canadian Centre for Mapping and Earth Observation) and Environment and Climate Change Canada will be responsible for the user segment, with the Canadian Forest Service leading the development and dissemination of the decision-support data products and knowledge exchange efforts. The Government of Canada has committed approximately $170 million towards the development of WildFireSat [32]. The cost schedule for WildFireSat (Canadian Space Agency, personal communications) is front end loaded, with most of the total budget devoted to pre-launch activities (satellite design and construction) and launch. The cost estimates used here have been varied ± 40% to allow for potential increases and decreases in costs, resulting in a range of values between $109 and $253 million (undiscounted).

### Impacts of WildFireSat

Simon et al. [16] employ microeconomic theory and Bayesian decision trees to illustrate how a decrease in uncertainty associated with wildland fire management decision making can lead to more efficient outcomes in the form of reduced costs and net value changes associated with wildfires. Similarly, here we assume that the decision-support products will lead to more informed wildfire management decisions. Ideally these augmented decisions will generate more effective and hence efficient outcomes (e.g., greater real time situational awareness, increased number of initial fire attacks within appropriate response timelines and more informed management tactics, etc.) which may influence suppression related decisions, costs, and fire activity. There is ample evidence suggesting that decreasing the uncertainty within wildland fire management decisions leads to more desirable outcomes (e.g., decreased wildfire related losses, improved safety, optimization of suppression resource allocation, etc. [14,33,34]). Changes in fire activity would directly affect timber, property, asset and infrastructure losses, evacuations, and the quantity and type of wildfire smoke released into the atmosphere, and many other societal issues. Our analysis focused on five cost components–for which data were available–affecting multiple stakeholders: (1) wildfire suppression costs (variable and fixed costs); (2) volumes of merchantable timber lost to wildfire; (3) property, asset and infrastructure losses (e.g., homes and businesses, power lines, roads, etc.); (4) evacuation costs for communities; (5) smoke-related health costs. We evaluated WildFireSat from a holistic perspective, noting that benefits may accrue to many groups (e.g., increased efficiency and/or decreased wildland fire suppression costs may manifest as lower government budgetary expenditures for management agencies, decreased insurance payouts may have business benefits, reduced evacuation avoid decreases in wellbeing, etc.).

**Fig 1:**
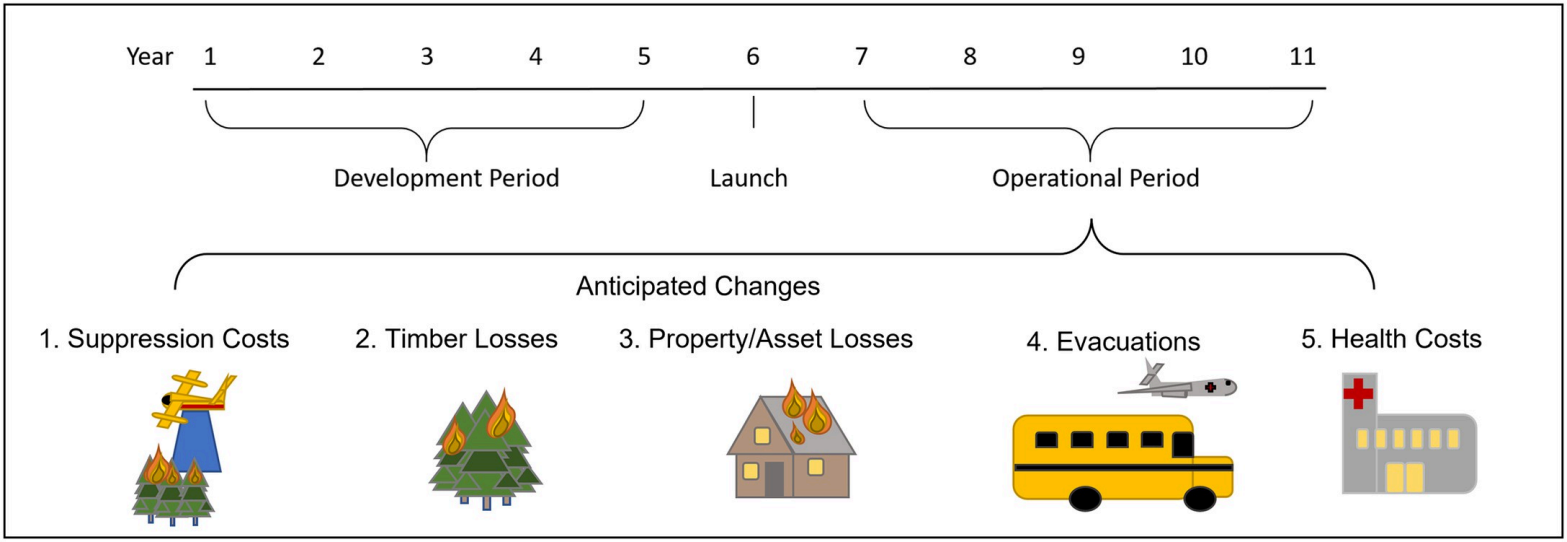
Anticipated key cost categories impacted by WildFireSat, once operational.

**Fig 2:**
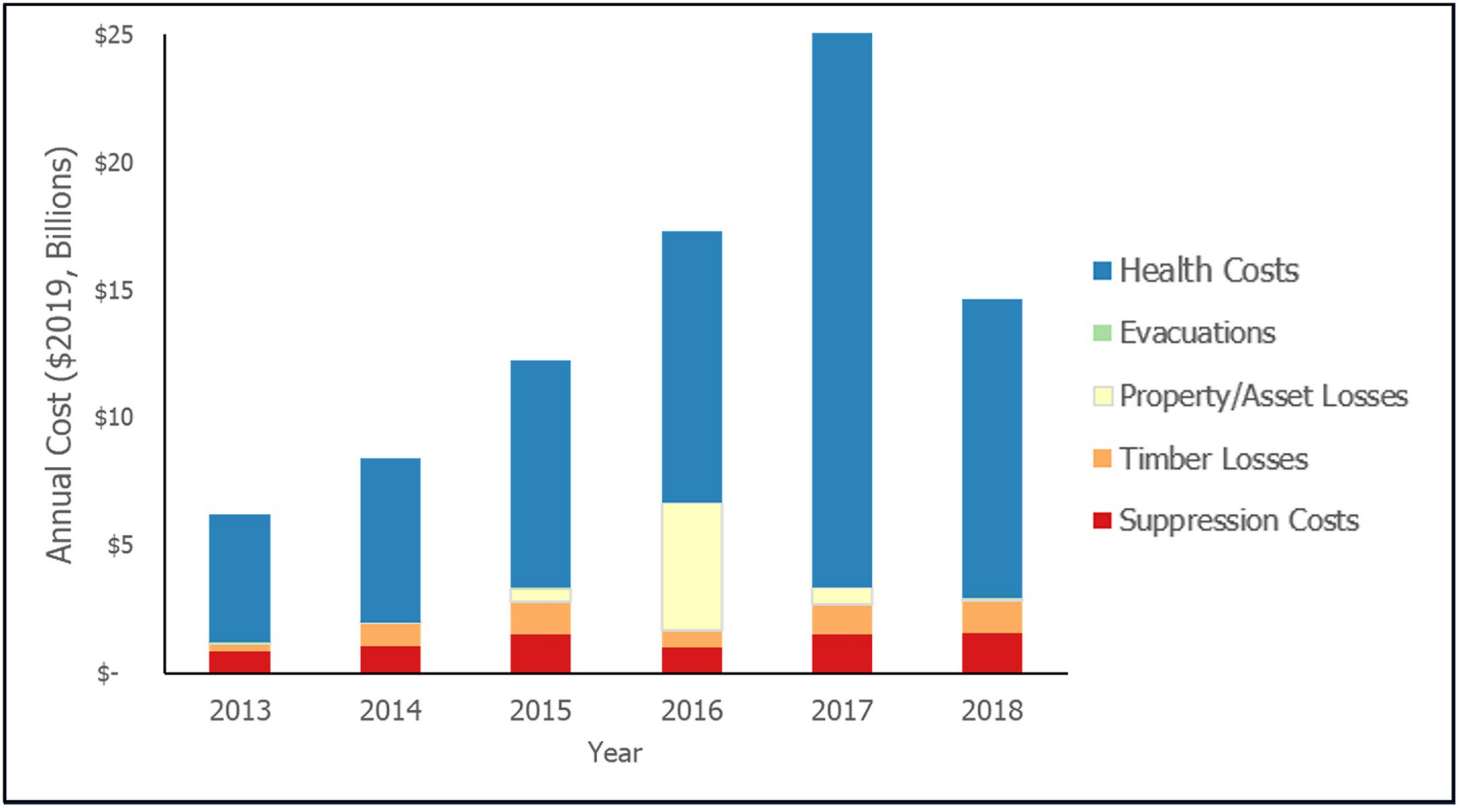
Cost component values over the 2013–2018 period.

The most comprehensive estimates for these cost components cover the 2013–2018 period; more recent data were unavailable. We used these data to develop a counterfactual narrative on what could have happened, had WildFireSat been operational over that timeframe and used to address fire activity during that period. This implicitly assumes the satellite would have been developed over the 2008–2012 period and operational in 2013 (see Fig 1). Table 1 describes the datasets and sources, and the calculations made to transform the available data into appropriate annual cost estimates for each component. Fig 2 illustrates the relative cost of each component during the 2013–2018 period.

**Table 1:** Description of the datasets used to form each cost category, an assessment of the confidence associated with the dataset/transformations, and dataset sources.

Dataset	Comments	Source
Suppression Costs	Dataset includes fixed and variable costs. Fixed costs cover organizational needs, including equipment maintenance costs, base employee pay, aircraft contract fees, etc. Variable costs address fire management needs including employee overtime pay, fuel costs, etc. Fire management agency cost data include all provincial and territorial agencies and Parks Canada.	Updated dataset provided by the Canadian Interagency Forest Fire Centre (CIFFC) upon request. Original dataset from [35].
Data Confidence	High; estimates provided by subject authority and no modifications required.	
Timber Losses	Data were based on calculations of fire emissions from the managed forest from 1990 to 2018, excluding non-forested lands (grasslands, wetlands, etc.) [36]. We assume that tree biomass is approximately 50% carbon [37] and derive a rough estimate of the total dry volume of tree biomass consumed by wildfire. We partitioned total biomass into the softwood (66%) and hardwood (34%) species proportions used by [30]. Using approximate merchantable volume to biomass volume conversion estimates (we assume between 70% and 90% of the biomass is merchantable using an identical uniform distribution for both softwoods and hardwoods) and volume to cubic meter conversions (softwoods: 1.82, hardwoods: 1.43 [38]), we calculate the volume of merchantable timber consumed by wildfires on an annual basis. The economic losses were estimated as the difference between the stumpage values the provinces/territories would have received had the fire not occurred, and the salvage value expected from post-fire logging. We presume that the amount salvaged ranges from 0% to 50%, right skewed to emphasize lower levels of salvage (W. Kurz, pers. com). A salvage volume cap of 50% was applied for ecological reasons [39]. Note that the estimates of timber losses do not account for impacts to wood harvesting companies; it is assumed that these organizations have the flexibility to shift their planned harvest to other areas, although they may choose to engage in removal of salvage wood.	Fire emissions: direct request to W. Kurz, CFS Senior Research Scientist, Carbon Accounting Team, at the Pacific Forestry Centre. See [36] for the methodology used to derive emission estimates. Merchantable volume to total biomass volume estimates based on [40–42] Provincial stumpage and salvage values: data collected from provincial websites and publications.
Data Confidence	Low; significant modification necessary to estimate economic value of timber lost to wildfire, excluding salvage amounts.	
Property, Asset and Infrastructure Losses	The Insurance Bureau of Canada only gathers data on larger scale catastrophic events that cost insurers over $25 million; wildfire-caused damage exceeded this limit five times between 1983 and 2017. We combined catastrophic fire insurance data with an estimate of the number of losses (primary and secondary residences, business losses, etc.) in those five years to estimate a high, medium and low insurance claim value per structure, which was used to parameterize a distribution of possible value per structure estimates. Randomly drawing a value per structure estimate from this distribution, we applied it to the number of structures lost to fires not within the Insurance Bureau’s database. These annual estimates were summed to create an estimate of total insurance claims over the period of interest. Uninsured losses are excluded from this approach; we assume uninsured losses to be about 2/3^rds^ of insured losses and inflate total losses by this amount to capture uninsured losses.	Insurance data collected from [43]. Dataset of wildfire evacuation events and counts of property losses originally developed by [44] and updated/maintained by the Canadian Forest Service 2021 Fraction of uninsured losses based on [45,46].
Data Confidence	Low; significant modifications required to derive estimates, and value per structure metric may not be representative in all cases.	
Evacuation Costs	Evacuation cost data are limited; evacuations of First Nations are typically funded by the federal government, while evacuations of non-First Nations are generally funded by provincial governments. Cost recovery data for First Nation evacuations were collected for the 2004–2020 period. These costs include payments made by the federal government to service providers (e.g. transportation, lodging, support, etc.), and to evacuees, compensating them for losses (e.g. food, clothing, health and wellness supports, property damage, etc.). Final cost estimates may take some time to compile; annual totals may reflect costs incurred in previous years. These values are likely to be an underestimate of total evacuation costs given the focus on Federal government costs, excluding all other costs incurred by provinces and communities.	Dataset of wildfire evacuation events and counts of property losses originally developed by [44] and updated/maintained by the Canadian Forest Service 2021. Evacuation cost recovery data from Indigenous Services Canada, provided on request. Details on First Nations evacuations and the role of the federal government available from [47].
Data Confidence	Low; although data provided by subject authority, known issues with data collection and timing, and unable to capture evacuation costs paid by other parties.	
Health Costs	Data includes national acute and chronic health costs, covering mortality and morbidity resulting from wildfire smoke. The dataset does not include health cost estimates for 2016; we use a linear projection from 2013–2015 to populate 2016 but acknowledge that this is likely an underestimate of health costs, given the wildfires that impacted Fort McMurray, Alberta that year.	Data derived from [48].
Data Confidence	Medium; moderate modifications required, acknowledged underestimate.	
WildFireSat Mission Costs	Annual cost estimates for WildFireSat, including pre-launch development costs, launch costs, and operating, maintenance and data downlink costs over the lifetime of the satellite. Includes a 40% contingency fee to cover unexpected costs.	Estimates provided by the Canadian Space Agency on request.
Data Confidence	High; data provided directly from subject authority; no modifications necessary.	

Although wildfire and wildfire-related impacts have become major areas of scientific inquiry [49], data and analyses quantifying the economic impacts of wildfire are limited. WildFireSat will likely generate impacts in other wildfire-related cost components beyond those captured here (e.g. effects on downstream businesses, watersheds and drinking water, well-being of communities impacted by wildfires, etc.), but data scarcity and difficulty quantifying these impacts prevents them from being included in this analysis.

To evaluate the potential scope of the impacts of WildFireSat *ex-ante*, we relied on expert opinions to inform our understanding of how wildfire management might be influenced by WildFireSat decision support data products. Because existing wildland fire management decision support products are not readily comparable to those anticipated from WildFireSat, collecting expert opinions on the possible outcomes associated with WildFireSat allowed for a more nuanced understanding of the positive and negative impacts the satellite mission could have. We interviewed five wildland fire management subject matter experts during the first quarter of 2021. At this time, WildFireSat was not yet formally publicized by the Government of Canada, hence we restricted our pool of experts to a group of five individuals with some prior knowledge of the mission.

All interviewees were presented with a slide deck outlining the authors’ understanding of how WildFireSat decision support products would fit into the existing wildland fire decision making system, and a set of descriptions and questions related to wildland fire management scenarios that might be influenced by WildFireSat decision support data products (e.g. how might these products be used to manage fires in remote locations or large project fires vs. daily decision making, etc.). Our interviews all followed a similar format: following a brief review of WildFireSat and the anticipated decision support data products, we discussed the expert’s understanding of how these products would fit into current decision-making processes, and the possible impacts of changes in decision making due to the satellite mission.

Opinions about WildFireSat varied considerably; some experts felt that WildFireSat decision support data products would make a considerable difference within wildland fire management, while others felt that the products were unlikely to lead to novel decision outcomes. Drawing on conclusions from these interviews and our own understanding of the possible impacts WildFireSat decision support data products could have on wildland fire management and downstream cost components, we parametrized four different impact distributions for use in the Monte Carlo analysis. All of our distributions are based on the PERT (Project Evaluation and Review Techniques) distribution [50], with variation driven by estimates of the maximum, minimum and most likely effects of the satellite decision support data products on the cost components. PERT distributions are often considered appropriate for representing uncertainty and capturing non-linear expert opinion [50]. They are preferred over triangular distributions for their computational simplicity, smoothness, and tendency to apply more weight to the most likely values (instead of the defined maxima and minima) [51].

**Fig 3:**
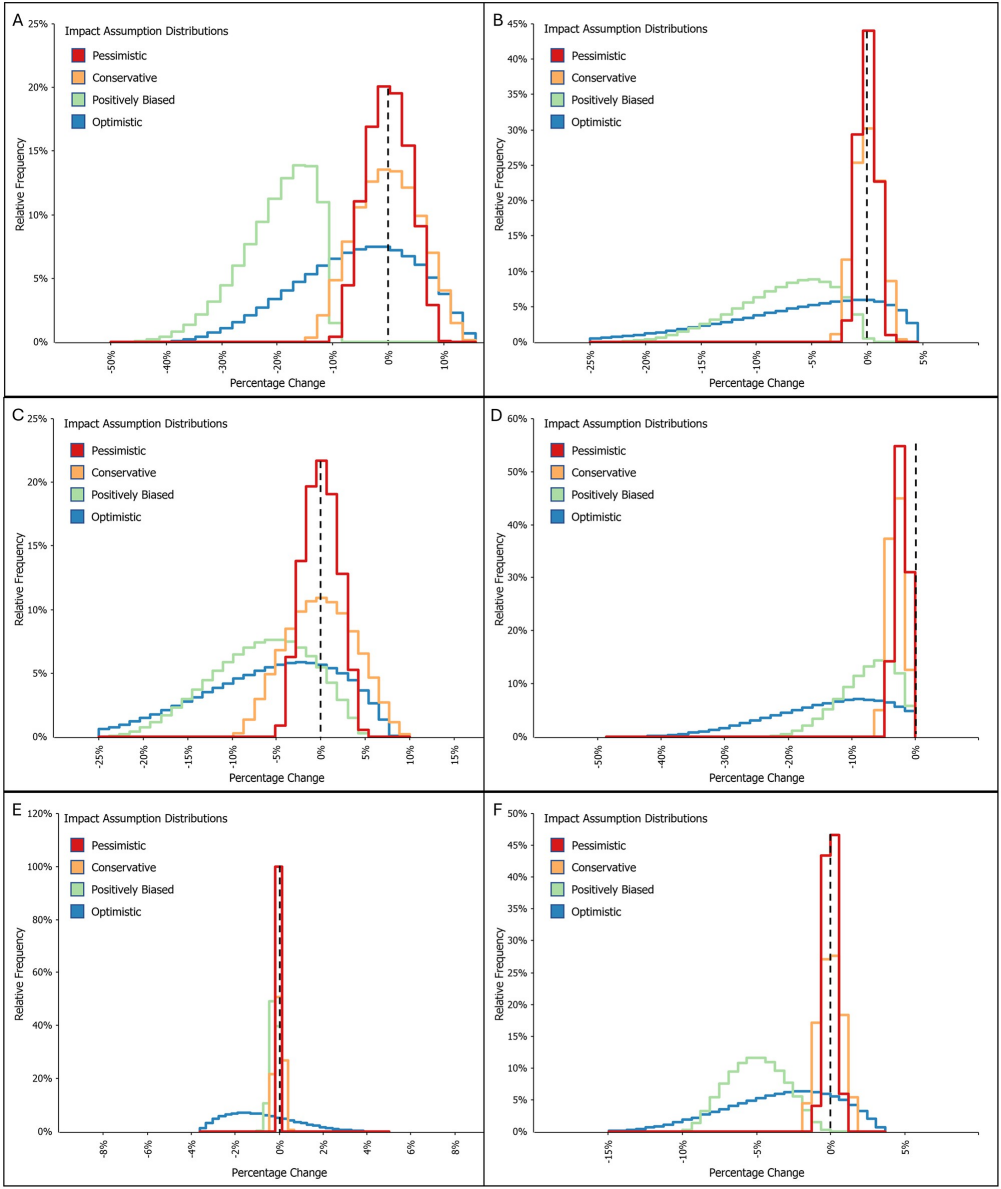
Simulated distributions of impacts assumptions (percentage change) for each cost component a) Suppression costs, variable, b) Suppression costs, fixed, c) Timber losses, d) Property, asset and infrastructure losses, e) Evacuation costs, f) Health costs. Note that the dashed line within each panel represents a 0% change in cost component value (i.e., the case without WildFireSat).

To simplify interpretation of the results, we categorize these distributions based on their shape: symmetric versus skewed (Fig 3a–3f). The symmetric and skewed distributions differ in shape, truncation points, and parameterization method (Tables 2 and 3). The more symmetric distributions are based on a modest expectation of how WildFireSat might affect fire management. They are likely appropriate for decision-makers inclined to believe that WildFireSat products would have a limited effect on outcomes. This could be driven by uncertainty as to the type of impact (either positive or negative) and potential barriers to adoption. There are a variety of barriers (e.g., distrust or a lack of confidence in satellite decision-support data products, preferences for other data types, delayed adoption, etc.) to successful implementation of innovations in fire management [52] that may restrict the integration of WildFireSat decision-support data products into existing management practices, reducing the resulting impacts. We explored two symmetric distributions: a narrower distribution, characterized by a tighter shape (hereafter referred to as the pessimistic distribution), and a wider distribution, representing a broader range of possible outcomes, with somewhat longer tails. While conservative, this distribution allows for a larger range of positive (cost decreases) and negative (cost increases) results.

**Table 2:** Assumed distribution parameters for all cost categories.

Cost Component	Distribution Details	Pessimistic	Conservative	Positively Biased	Optimistic
Variable Suppression Costs	Min	-10%	-15%	-50%	-50%
	Mean	0%	0%	-15%	-5%
	Median	0%	0%	-19%	-5%
	Max	10%	15%	-10%	25%
	Skewness	0.00	0.00	-0.76	-0.37
	Kurtosis	2.33	2.33	3.11	2.33
Fixed Suppression Costs	Min	-2%	-3%	-25%	-25%
	Mean	0%	0%	-5%	-5%
	Median	0%	0%	-7%	-5%
	Max	2%	3%	0%	5%
	Skewness	0.00	0.00	-0.58	-0.62
	Kurtosis	2.33	2.33	2.78	2.63
Timber Losses	Min	-5%	-10%	-25%	-25%
	Mean	0%	0%	-5%	-5%
	Median	0%	0%	-6%	-5%
	Max	5%	10%	5%	-8%
	Skewness	0.00	0.00	-0.30	-0.40
	Kurtosis	2.33	2.33	2.45	2.41
Property, Asset & Infrastructure Losses	Min	-5%	-6%	-25%	-50%
	Mean	-2%	-3%	-7%	-12%
	Median	-2%	-3%	-7%	-13%
	Max	0%	0%	0%	0%
	Skewness	-0.18	0.00	-0.58	-0.59
	Kurtosis	2.38	2.33	2.78	2.73
Evacuation Costs	Min	-1%	-2%	-10%	-15%
	Mean	0%	0%	-5%	-3%
	Median	0%	0%	-5%	-3%
	Max	1%	2%	0%	5%
	Skewness	0.00	0.00	0.00	-0.42
	Kurtosis	2.33	2.33	2.33	2.56
Health Costs	Min	-0.01%	-0.5%	-1%	-5%
	Mean	0%	0%	-0.2%	-1%
	Median	0%	0%	-0.2%	-1%
	Max	0.01%	0.5%	0.0%	5%
	Skewness	0.00	0.00	-0.83	0.51
	Kurtosis	2.33	2.33	3.26	2.68

**Table 3:** Description of the parametrization of the distribution of WildFireSat impacts.

**Variable Suppression Costs**	
WildFireSat data will likely influence suppression decisions. This could result in changes to variable suppression costs (e.g., fire crew overtime wages, additional short-term fire crew and aircraft hires, costs of borrowing resources, etc.,[30]). The impacts on variable suppression costs are likely to be moderate.	
Pessimistic and Conservative	Moderate changes (increase or decrease) in variable suppression costs are expected to be equally likely. These moderate level impacts are more likely if WildFireSat data are not well integrated into suppression decisions or produces decisions identical to those that would have been made without the satellite data.
Positively Biased	Large to moderate decreases in variable suppression costs are expected. Larger decreases are expected if WildFireSat data are highly integrated into decision making, and the novel data lead to substantial changes in decision outcomes that reduce/optimize suppression efforts.
Optimistic	Although a decrease in variable suppression costs is expected, increases in costs are possible. Assuming well integrated WildFireSat data, variable suppression costs could decrease if suppression efforts are reduced/optimized. Alternatively, costs could increase if the satellite data support increased suppression activities.
**Fixed Suppression Costs**	
WildFireSat data may have some impact on fixed suppression costs (e.g., hiring and training fire crews, maintaining aircraft and other fixed infrastructure, etc.) over the short- and long-term, but the magnitude of the impacts are likely to be small.	
Pessimistic and Conservative	Small changes in fixed suppression costs (increases or decreases) may be equally likely. If WildFireSat data are used to justify modifications to contracted resources and employee structures, which could result in small increases or decreases in fixed suppression costs.
Positively Biased	Moderate to small decreases in fixed suppression costs are expected. Well integrated and trusted WildFireSat data may replace a large proportion of the intelligence gained from aerial monitoring, avoiding some costs related to aircraft contracts or reducing demands on existing aircraft resources. Evidence collected from WildFireSat data may also be used to support larger, cost-saving organizational changes (e.g., reduced monitoring budget) through time.
Optimistic	Moderate to small decreases in fixed suppression costs are expected, although small increases in costs are possible. Although WildFireSat data products integrated into decision making may lead to changes in aerial monitoring costs and larger organizational changes, these data products may also highlight a need for specific suppression resources, personnel, or novel organizational structures, increasing fixed suppression costs over time.
**Timber Losses**	
Decision making supported by WildFireSat data products may impact area burned, and the resulting timber losses, producing decreases if fires are suppressed quickly, or even increases if more fires are allowed to burn on the landscape. The impacts on timber losses are more likely to be moderately sized.	
Pessimistic and Conservative	Moderate to small changes in timber losses (increases or decreases) are expected to be equally likely. WildFireSat data products may result in minor impacts on timber losses if the data products are not well integrated into decision making or if WildFireSat data does not support changes to current decision making.
Positively Biased	Moderate to small decreases in timber losses are expected, although small increases in losses may occur. Well-integrated WildFireSat data products may support fire management decisions that significantly reduce area burned and the associated timber losses. WildFireSat data may also support decisions that allow for more fire on the landscape, potentially increasing the amount of timber burned.
Optimistic	Moderate to small decreases in timber losses are expected, although moderate increases in losses may occur. WildFireSat data are more likely to support decisions that reduce area burned but may also support fire management decisions that allow more fire activity on the landscape as part of various landscape management objectives.
**Property, Asset & Infrastructure Losses**	
As WildFireSat data affects suppression effectiveness and timber losses (via area burned), it will also affect property, asset and infrastructure losses. An increase in losses as a result of WildFireSat data is unlikely; data products providing landscape level information are more likely to encourage property, asset and infrastructure protection. The size of the impact on property asset and infrastructure losses varies, depending on assumptions.	
Pessimistic and Conservative	Small decreases in property, asset and infrastructure losses are expected. If WildFireSat data products are not well integrated into decision making, then the impacts on suppression effectiveness and subsequently property, asset and infrastructure losses may be minimal. Alternatively, WildFireSat data may support decision outcomes unchanged from current decisions.
Positively Biased	Moderate to small decreases are expected. WildFireSat data products integrated into the decision-making process are likely to lead to more effective suppression decision outcomes, property, asset and infrastructure losses could be decreased significantly.
Optimistic	Large to moderate decreases in property, asset and infrastructure losses are expected, if WildFireSat data products supported decisions that improved the ability of fire management agencies to suppress fire across multiple factors (e.g., improved pre-fire allocation of resources, optimized fire suppression, etc.)
**Evacuation Costs**	
By affecting suppression effectiveness, WildFireSat data will also indirectly affect evacuations (and their associated costs), which may be impacted by improved fire behaviour modelling or smoke forecasting. The size of the impact on evacuation costs varies, depending on assumption.	
Pessimistic and Conservative	Small changes in evacuation costs (increases or decreases) may be equally likely. Changes to evacuation costs may be minimal if WildFireSat data are not well integrated into decision making, or decisions supported by WildFireSat lead to similar outcomes.
Positively Biased	Moderate to small decreases in evacuation costs are expected. Cost reductions may be possible if evacuations can be avoided or shortened via WildFireSat data-supported suppression, or improved fire behaviour modelling.
Optimistic	Moderate to small decreases in evacuation costs are expected, but small increases in evaluation costs are possible. Fire management or evacuation decisions informed by WildFireSat data may lead to avoided/shortened evacuations, thereby reducing costs. Alternatively, evacuation costs could increase if WildFireSat data supports increasing the number and/or length of evacuations.
**Health Costs**	
WildFireSat data will similarly affect health costs via smoke impacts, which will be affected by changes in suppression effectiveness and timber losses (area burned). The magnitude of impact is likely to be minute, given the atmospheric transport of wildfire smoke from jurisdictions outside of Canada, and the scale of the impacted population.	
Pessimistic and Conservative	Minute changes in health costs (increase or decrease) may be equally likely. Health costs may not be impacted using WildFireSat data, if the data products are not well integrated, or lead to similar decision outcomes.
Positively Biased	Small changes in health costs are expected. Well-integrated WildFireSat data may support increased suppression, or targeted suppression of fires posing a smoke threat, reducing health costs.
Optimistic	Small changes in health costs are expected, but small increases may also be possible. WildFireSat informed decisions that lead to decreased fire activity may result in less smoke or reduced exposure to smoke, decreasing health costs. Alternatively, if the data suggests that more fires should be monitored instead of suppressed, airborne smoke and related health costs may increase.

The skewed distributions are parametrized based on perspectives regarding the use of WildFireSat decision-support products within specific wildfire situations (e.g., circumstances with scarce suppression resources or limited fire intelligence data, etc.). The long left-side tails reflect an increased possibility of positive, cost-reducing impacts. These scenarios are intended to represent decision-makers with stronger, positive opinions regarding the impacts of WildFireSat, or more optimistic beliefs about the adoption and integration of WildFireSat products into decision-making processes. We evaluated two skewed distributions: a left-skewed distribution and a less-skewed distribution. The left-skewed positively biased distribution represents strong, positive WildFireSat-related impacts and assumes limited potential for negative impacts. The less-skewed optimistic distribution represents a more nuanced view, incorporating smaller negative and larger positive impacts resulting from WildFireSat. These two distributions differ in shape and assumed most likely values. These distributions were presented and discussed at the 2022 International Wildland Fire Canada Conference [53] for feedback; the audience was in general agreement with the distributions and ranges of values they captured.

Importantly, many of our impact assumption distributions allow for both positive and negative effects. Discussions with experts suggested that WildFireSat-supported decisions could result in decreases *and* increases in fire activity in various situations (WildFireSat Development Team, personal communication). While decision outcomes that reduce fire activity are thought to be more likely, fire activity may also increase, depending on each wildfire’s unique management objectives and policies faced by the decision-maker and their opinions about unquantified market and non-market values. For example, instead of the traditional ‘full response’ where fires are actively suppressed, some situations may permit a ‘modified’ or ‘monitored’ approach [9], with the expectation that the overall outcome across *all* fire management objectives (e.g., protection of human life, asset protection, and maximizing expected benefits of fire on the landscape) would be positive. WildFireSat decision-support data products may allow for more frequent and effective application of these kinds of more nuanced suppression policies resulting in an increase in fire activity where more fire on the landscape is desirable [10,54]. There are challenging tradeoffs between competing objectives and varying decision-maker risk tolerances about perceived impacts [55].

### Analysis time frame

WildFireSat design and construction costs are expected to commence five years prior to satellite launch. Once launched, the mission will continue to incur operational costs, but will also begin producing benefits. Our model accounts for both the design and operational phases, calculating mission costs and benefits over the 2007–2018 period. As noted, 2013–2018 data are intended to represent a period WildFireSat decision-support data products would have been available, requiring the assumption that the design phase began in 2007. Costs are calculated over the lifetime of the satellite mission, while benefits are estimated as the value of avoided losses only between 2013–2018. The net present value subtracts the benefits from the mission costs, using a 6% discount rate (although this is adjusted in subsequent sensitivity analyses described below). The discount rate allows us to account for the economic implications associated with the timing of WildFireSat costs and benefits; 6% is a compromise between the lower rates occasionally used by governments evaluating natural resource investments [56,57] and the higher rates recommended by some for public policy decisions [58]. Final results are inflated to 2019 values, using the Canadian Price index [59].

### Sensitivity analysis

In addition to exploring the effect of different distributions on the results, we investigated the implications of other model assumptions via sensitivity analyses. Adjusting and testing the parameter values within our model lends additional credibility to the selected values, but also increases confidence in the model outputs. We examined the following parameters: 1) changing the discount rate; 2) excluding the health cost component; 3) expanding mission costs to account for unforeseen cost overruns and launch delays; 4) adjusting the model to allow correlation between impact assumption distributions. We iterated the model 50,000 times for each component of the sensitivity analysis, after which the model again achieved stability.

## Results and discussion

The estimated median present value of the cost of wildfire in Canada over the study period, without the effects of WildFireSat, is $57,220 million (standard deviation: $3,407 million; Table 5). This value represents the sum of the cost components over the 2013–2018 period, deflated to 2007, and then inflated to 2019 values. Health costs account for 75% of these costs on average, annually. The remainder is divided across the other components: 10% attributed to suppression costs, (7% variable, 3% fixed costs), 9% attributed to timber losses, 6% to property, asset and infrastructure losses and less than 1% attributed to evacuation costs (Table 4).

**Table 4:** Cost component estimates, in millions ($2019, CAD), 90% confidence intervals and median present values (in brackets).

Scenarios	Cost Component	Sum of each Component over five Years			
Without-WildFireSat	Variable Suppression Costs	$3,469–$3,469 ($3,469)			
	Fixed Suppression Costs	$1,566–$1,566 ($1,566)			
	Timber Losses	$1,389–$10,538 ($4,634)			
	Property, Asset & Infrastructure Losses	$2,668–$6,270 ($4,198)			
	Evacuation Costs	$61.01–$61.01 ($61.01)			
	Health Costs	$43,095–$43,095 ($43,095)			
Distributions		Pessimistic	Conservative	Positively Biased	Optimistic
With-WildFireSat	Variable Suppression Costs	$3,426–$3,513 ($3,469)	$3,405–$3,534 ($3,469)	$2,931–$3,418 ($3,229)	$2,814–$3,574 ($3,301)
	Fixed Suppression Costs	$1,468–$1,663 ($1,566)	$1,420–$1,712 ($1,566)	$1,056–$1,386 ($1,271)	$1,174–$1,722 ($1,487)
	Timber Losses	$1,386–$10,527 ($4,628)	$1,387–$10,572 ($4,623)	$1,284–$9,861 ($4,317)	$1,297–$10,001 ($4,348)
	Property, Asset & Infrastructure Losses	$2,610–$6,139 ($4,106)	$2,589–$6,079 ($4,072)	$2,450–$5,828 ($3,882)	$2,209–$5,540 ($3,597)
	Evacuation Costs	$60.03–$61.39 ($61.01)	$60.25–$61.77 ($61.01)	$56.06–$59.85 ($57.96)	$54.92–$62.23 ($59.18)
	Health Costs	$43,093–$43,098 ($43,095)	$42,961–$43,229 ($43,095)	$42,862–$43,082 ($43,008)	$41,802–$43,957 ($42,664)

Incorporating WildFireSat impacts over the same five-year period reduces the median present value of the total cost of wildfire to between $55,721 and $57,122 million (Tables 4 and 5). Note that the more extreme values come from the conservative and positively biased impact distributions; the slightly skewed nature of some of the distributions pushes the median value of these distributions lower/higher than the pessimistic and optimistic impact distributions. The symmetric distributions result in relatively smaller WildFireSat-related impacts, producing cost estimates closer to those reported in the without-WildFireSat scenario (pessimistic: median NPV of $57,122 million, standard deviation of $3,399 million; conservative: median NPV of $57,085 million, standard deviation of $3,405 million). The skewed distributions generated larger, more positive-leaning impacts, and produced smaller cost estimates (positively biased: median NPV of $55,908 million, standard deviation of $3,229 million; optimistic: median NPV of $55,721 million, and standard deviation of $3,326 million).

**Table 5:** Calculation results across all distributions, 90% confidence intervals with median present values in brackets, in millions ($2019, CAD).

Distributions		Pessimistic	Conservative	Positively Biased	Optimistic
5-year Cost Component Sum	Without WildFireSat	$52,775–$63,747 ($57,220)			
	With WildFireSat	$52,704–$63,622 ($57,122)	$52,656–$63,627 ($57,085)	$51,660–$62,105 ($55,908)	$51,300–$62,009 ($55,721)
WildFireSat Benefits		$-126.9–$317.4 ($92.4)	$-295.3–$554.1 ($126.1)	$692.4–$2,208 ($1,275)	$-25.71–$3,111 ($1,534)
Satellite Mission Costs		$110.0–$182.8 ($146.4)			
Benefit—Cost Estimate (NPV)		$-276.3–$174.2 ($-54.2)	$-441.9–$408.3 ($-19.85)	$545.3–$2,062 ($1,128)	$-172.1–$2,965 ($1,388)

Overall, we estimate the median gross benefit of the WildFireSat mission to be between $92.4 million and $1,534 million, depending on the distribution of assumptions used, noting that WildFireSat mission costs have not yet been incorporated into the estimate (Table 5). The symmetric distributions produce lower benefit estimates (pessimistic: median NPV of $92.4 million, standard deviation of $137.27 million; conservative: median NPV of $126.1 million, standard deviation of $264.2 million), and the skewed, more positive distributions produce larger estimates (positively biased: median NPV of $1,275 million, standard deviation of $476.4 million; optimistic: median NPV of $1,534 million, standard deviation of $957.1 million).

Combining these avoided losses/benefits with the discounted median mission cost estimate of $146.4 million produces a range of NPVs: -$54.2 million (standard deviation: $139.0 million) and -$19.85 million (standard deviation: $265.2 million) for the pessimistic and conservative symmetric distributions and $1,128 million (standard deviation: $477 million) and $1,388 million (standard deviation: $957. 1 million) for the positively biased and optimistic skewed distributions (Table 5). The distributions of NPV results for all impact assumption distributions are unimodal, and relatively symmetric about their means. They generally reflect the input assumption distributions; the symmetric distribution results are comparatively narrow with shorter tails, while the skewed distributions are much more widely distributed with relatively long tails.

**Fig 4:**
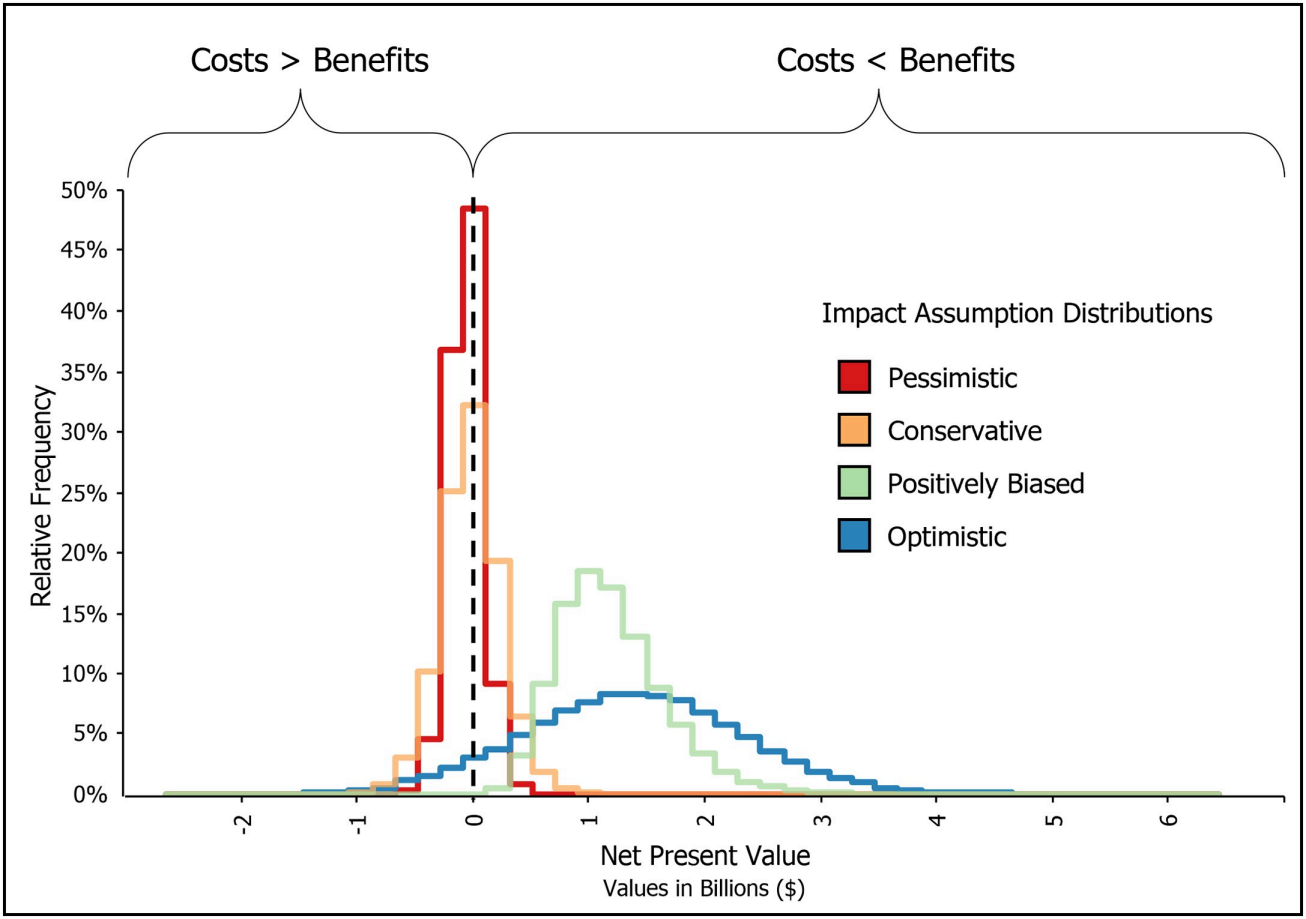
Results distributions, illustrating the Net Present Value (NPV) for each set of impact assumption distributions. A negative value indicates costs exceed benefits, and a positive value indicates benefits exceed costs.

These NPV results indicate that large positive economic benefits are possible, depending on assumptions about the use of the new satellite data and adoption levels, which will support a positive net result. Under the skewed impact assumption distributions, the median value of the benefits varies between 8.72 and 10.48 times larger than mission costs (optimistic and positively biased scenarios, respectively). Under the symmetric distributions, negative outcomes are more likely, suggesting that the model-forecasted wildfire management-related benefits are insufficient to offset the development costs in those scenarios. In these cases, mission costs are between 1.59 and 1.16 times larger than the estimated benefits for the pessimistic and conservative scenarios, respectively. Fig 4 illustrates the distributions for each NPV outcome. The width and skewedness of the outcome distributions are highly dependent on the four feasible expert-driven impact assumption distributions. More than 50% of the model iterations produce negative NPVs under the symmetric distributions (67% and 53% for the pessimistic and conservative symmetric distributions, respectively). Negative NPVs are far less likely under the skewed distributions. In these cases, the model produces positive NPVs for most iterations (93% and 100% of the iterations for the optimistic and positively biased distributions, respectively).

The assumptions embedded in the various scenarios represented here should be viewed as providing insights into the net worth of the mission from a wide range of perspectives. Some impact assumption distributions may be more likely to occur, according to some stakeholders. Capturing this range of perspectives is important given the stochastic and uncertain nature of this analysis. The WildFireSat mission includes a strong focus on knowledge exchange, which is intended to encourage awareness and uptake of the resulting decision-support data products by end users [60]. This knowledge exchange investment may be critical to ensure that the skewed, positive-leaning distributions are more likely to be achieved.

### Sensitivity analysis results

We selected four variables for the sensitivity analysis within the model subject to the greatest uncertainty. First, we varied the discount rate; there are highly varying views about appropriate rates for public investments [57]. Second, smoke-related health costs represent a large proportion of the wildfire-related impacts examined but are subject to high levels of uncertainty (origin, delayed onset of health problems, variation in estimating techniques, etc.). Third, accounting for shifts in mission costs (i.e., launch delays and cost overruns) is prudent, given how common these adjustments are within the satellite sector. Finally, evaluating the effect of correlative impacts between the cost components allows us to examine model outcomes under more nuanced conditions (as described below).

Table 6 illustrates the median and 90% confidence interval values for these selected sensitivity analysis variables. Despite stressing appropriate extremes of these variables, the conclusions drawn from the sensitivity analyses remain generally unchanged from the base case. The pessimistic and conservative assumptions lead to lower and occasionally negative NPV outcomes, while the positively biased and optimistic assumptions always produce positive outcomes. It is possible that the existing variation built into the model is much larger than that introduced by the sensitivity analysis, such that stressing values within the sensitivity analysis fails to noticeably impact model NPV outcomes. Given this outcome from the sensitivity analysis, it is likely that the most important model component is the selection of the impact distributions; they are the primary driver of model results.

**Table 6:** Calculation results across each sensitivity analysis simulation, 90% confidence intervals with median NPVs in brackets, expressed in millions.

Distributions		Pessimistic	Conservative	Positively Biased	Optimistic
Discount Rate	0%	$-403.8–$346.0 ($-34.78)	$-682.6–$742.1 ($23.94)	$964.1–$3,513 ($1,942)	$-245.0–$5,061 ($2,401)
	2%	$-353.9–$276.4 ($-43.58)	$-586.9–$607.4 ($5.45)	$794.9–$2,930 ($1,615)	$-215.5–$4,217 ($1,994)
	4%	$-311.8–$219.9 ($-49.94)	$-507.7–$497.6 ($-8.84)	$657.4–$2,454 ($1,347)	$-190.7–$3,529 ($1,661)
	8%	$-246.4–$137.2 ($-57.14)	$-386.6–$335.8 ($-28.18)	$453.1–$1,739 ($947.7)	$-156.2–$2,499 ($1,164)
	10%	$-220.4–$107.3 ($-58.78)	$-340.1–$276.1 ($-34.34)	$376.6–$1,471 ($797.9)	$-141.7–$2,113 ($979.0)
Exclude Health Costs		$-276.3–$174.1 ($-54.42)	$-422.4–$389.5 ($-19.84)	$454.1–$1,955 ($1,026)	$62.34–$2,289 ($962.8)
Mission Costs	50%	$-352.9–$103.8 ($-127.3)	$-517.3–$338.8 ($-93.01)	$470.7–$1,991 ($1,055)	$-245.3–$2,894 ($1,315)
	100%	$-431.4–$34.74 ($-200.4)	$-593.5–$268.8 ($-166.2)	$394.4–$1,920 ($982.4)	$-318.1–$2,823 ($1,243)
	150%	$-510.7–$-32.2 ($-273.8)	$-670.66–$198.2 ($-239.1)	$318.0–$1,850 ($910.1)	$-389.8–$2,749 ($1,169)
	Delay 1 Year	$-292.6–$141.8 ($-78.51)	$-451.7–$366.8 ($-45.51)	$496.8–$1,953 ($1,056)	$-193.3–$2,820 ($1,306)
	Delay 2 Years	$-307.6–$110.8 ($-101.4)	$-458.5–$326.5 ($-69.44)	$448.8–$1,845 ($985.7)	$-209.5–$2,678 ($1,226)
Correlation		$-349.1–$246.7 ($-55.25)	$-616.58–$586.7 ($-17.52)	$352.2–$2,330 ($1,109)	$-833.6–$3,679 ($1,379)

#### Discount rate

Given the time lag between costs and eventual impacts expected to accrue from WildFireSat, and the uncertainty regarding appropriate discount rates for government investments in projects with risky outcomes [61,62], we examined the effect of other discount rates (0%, 2%, 4%, 8% and 10%) beyond the base case discount rate of 6% (see Table 5 for base case results). Decreasing the discount rate to 0% increases the median NPV estimates for all scenarios; for the pessimistic scenario the median NPV estimate increases by 36%, moving from a base case value of $-54.2 million to $-34.78 million, while the median NPV estimate increases by 221% under the conservative scenario (a base case value of $-19.85 million increasing to $23.94 million). Similarly, the median NPV estimates increase for the positively biased scenario (72% increase, from a base case value of $1,128 million to $1,942 million) and the optimistic scenario (73% increase, from a base case value of $1,388 million to $2,401 million).

Reducing the discount rate to 2% or 4% also increases in NPVs above the base case values for all scenarios, but to a smaller extent. The pessimistic scenario median NPV increased by 20% under a 2% discount rate, resulting in a median NPV of $-43.58 million, and increased by 8% under a 4% discount rate, generating a median NPV very close to the base case value ($-49.94 million). The conservative scenario increases by 127% under a 2% discount rate (median NPV of $5.54 million), and by 55% under a 4% discount rate (median NPV of $-8.84 million). Under a 2% discount rate, the positively biased scenario increases by 43% ($1,615 million) and increases by 19% ($1,347 million) under a 4% discount rate. Similarly, the optimistic scenario increases by 44% ($1,994 million) under a 2% discount rate, or by 20% ($1,661 million) under a 4% discount rate. Increasing the discount rate beyond the base case value of 6% results in decreases in median NPV estimates for all scenarios. For the higher discount rates, the pessimistic scenario median NPV decreases by 5% or 8% under the 8% and 10% discount rates, respectively. These changes results in the similar median NPVs ($-57.14 to $-58.78 million). The conservative scenario estimate decreases by 42% or 73% under an 8% or 10% discount rate respectively, also producing similar median NPVs ($-28.18 to $-34.34 million). The positively biased scenario estimate decreases by 16% (8% discount rate, $947.7 million), or 29% (10% discount rate, $797.9 million), and the optimistic scenario estimates decrease by a similar amount (an 8% discount rate produces a 16% decrease, with a NPV of $1,164 million, while a 10% discount rate generates a 29% decrease in median NPV to $979.0 million).

Although lower discount rates (0%, 2% and 4%) result in higher NPV estimates, changing the discount rate did not change the overall net outcome of the analysis (i.e., distributions that produced positive (negative) NPVs under the base case discount rate continue to produce positive (negative) NPVs with lower or higher discount rates) for most of the impact assumption distributions. The conservative distribution is the exception; estimated NPVs are positive under lower discount rates (0% and 2%). This would suggest that conservative estimates about the impact of WildFireSat may produce positive benefits if lower discount rates are used, increasing the relative importance of the delayed anticipated benefits of WildFireSat above that assumed with higher discount rates.

#### Health cost component

The health costs attributed to wildfire smoke represent the largest proportion of the total value of the cost components examined. The magnitude of these impacts could suggest that fire suppression at any cost would be justified from a health perspective. Fire, however, is an important ecological component of Canadian forest systems. Complete elimination of fire is undesirable and untenable [63]. Management decisions must balance the health costs associated with smoke and other competing fire management objectives. Although the linkages between wildfire smoke, detrimental health impacts, and resulting economic costs are well established [48,64–66], attributing health impacts to specific fire seasons (and individual fires) is more problematic. Atmospheric transportation of smoke, cross-border smoke impacts, delayed health effects, and the size of the impacted population (whether near or far from a fire event) all make connecting specific fire events to explicit health impacts difficult.

Given this uncertainty, and the impact health costs could have on the results due to their magnitude, we explored a scenario that excluded health costs. Importantly, re-calculating NPVs without health costs produces a very small change in the net result. Relative to the base case, little change in median NPV is discernable for the symmetric distribution scenarios (median NPVs decrease by 0% for both the pessimistic and conservative distributions, staying around the median base case values of $-54.2 million and $-19.85 million, respectively), while a somewhat larger decrease occurs for the skewed distributions. For the positively biased distribution, median NPVs decreased from the base case values of $1,128 million by 9% to $1,026 million. For the optimistic distribution, median NPVs decrease 31% from the base case values of $1,3988 million to $962.0 million. These adjustments to NPV are a direct result of our assumptions about the impact WildFireSat could have on health costs. Under the pessimistic and conservative scenarios, we assume WildFireSat decision-support products will have a very limited impact on health costs (i.e., ± 0.01% and ±0.50% respectively). For the more optimistic scenarios (positively biased, and optimistic), decision-support products would produce slightly larger adjustments (increases or decreases) to health costs (i.e., ±1% and ±5% respectively). Hence, the effect of removing this depends on the size of the assumed impact.

#### Mission cost overruns and launch delays

Our initial analysis is based on the development, construction, and operational costs of the satellite constellation, as well as the costs associated with decision-support data product development and knowledge exchange efforts. Although provided cost estimates are likely robust, satellite projects are known to be subject to cost overruns and launch delays [67]. Additionally, cost forecasts associated with novel projects are often underestimated and uncertain [68]. Here we explore two distinct possibilities: a static increase in costs (that is, annual mission cost estimates are increased by 50%, 100% and 150%), and a 1- or 2-year delay in the project, pushing the launch date back. Historical satellite projects have suffered from costs overruns ranging from 25% to over 1,000% [67]; we feel that the range of overruns selected, plus the assumed 40% variation already built into the mission costs, is likely sufficient to capture unforeseen increases associated with these costs. For the delayed simulations, we assume a pre-launch cost schedule identical to the baseline simulation, with those costs expected in the year(s) prior to launch duplicated during the additional years.

Modifying WildFireSat’s mission cost estimates to include cost overruns and launch delays resulted in decreases in median NPV estimates for all distributions across all scenarios, with larger decreases observed across the symmetric scenarios. This impact on the symmetric distributions is a result of their narrow shape; limiting the variation within the assumption limits the variation in all subsequent calculations, affecting the distribution’s ability to absorb large cost increases without affecting the ultimate net result.

When mission costs exceed the original cost estimates by 50% to 150%, median NPV results decreased between 135% and 405% for the pessimistic distribution (decreasing from the median base case pessimistic value of $-54.2 million to between $-127.3 and $-273.8 million), and 369% and 1,105% for the conservative distribution (decreasing from the median base case conservative value of $-19.85 million to between $-93.01 and $-239.1 million). For the skewed distributions however, the proportional impact of including the cost overruns was relatively muted, with small decreases to median NPVs. For the positively biased distribution, median values decreased from the base case value of $1,128 million by 6% ($1,055 million) to 19% ($910.1 million). For the optimistic distribution, median values decreased from the base case of $1,388 million by 5% ($1,315 million) to 16% ($1,169 million).

Similarly, when we assume that launch has been delayed, median NPV results decrease by 45% to 87% (1- and 2-year delays, respectively) for the pessimistic distribution (resulting in values between $-78.51 and $-101.4 million), and 129% and 205% for the conservative distribution (values between $-45.51 and $-69.44 million). Delaying launch produced similar results for both skewed distributions, decreasing median NPVs by 6% (1-year delay; the median positively biased value decreased to $1,056 million, and the median optimistic value decreased to $1,306 million) and 12% (2-year delay; positively biased median NPV decreased to $1,306 million, and the optimistic median NPV decreased to $1,226 million). Unsurprisingly, increasing annual mission costs to account for possible cost overruns resulted in a larger impact than incorporating a launch delay of one (or two) years.

#### Cost component correlation

Although decisions informed by WildFireSat may lead to reductions in suppression costs, we make no assumptions about the subsequent direction of impact on timber losses, property, asset and infrastructure losses, evacuations, and health costs. Difficulty specifying the direction of the relationship (positive or negative) between the impact assumptions over the course of WildFireSat’s operational period led us to model independently distributed components within our estimates. In reality, a decrease (or increase) in suppression costs is likely correlated with changes in other cost components to some extent. A decrease in suppression costs could be correlated with an increase in timber losses if suppression expenses are avoided by allowing more fires to burn, which presumably results in the combustion of more forest fuels. Alternatively, a decrease in suppression costs could be related to a decrease in timber losses, if WildFireSat decision-support products lead to decisions that optimize suppression resource distribution, thereby reducing suppression costs *and* decreasing area burned.

**Fig 5:**
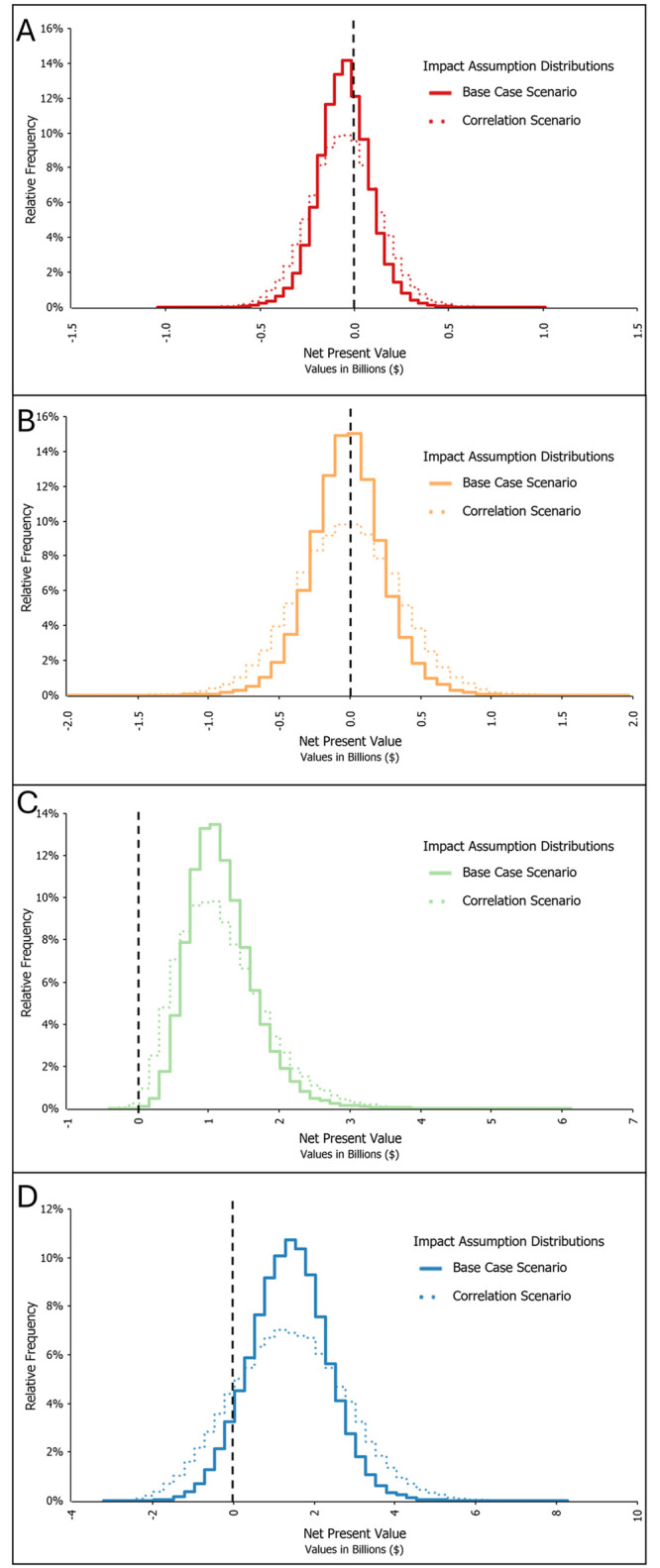
Correlation scenario results, by impact assumption distribution (a) Pessimistic, (b) Conservative, (c) Positively biased, (d) Optimistic.

Here we relax the assumption of independent distributions and presume that the impact assumptions distributions are correlated. Correlation values were based on coefficients calculated from the time series data available for each impact factor (see Table 7), and manually adjusted to reflect our beliefs about the causal effects of WildFireSat (e.g. the cost component time series data suggest a negative relationship between fixed suppression costs and property, asset and infrastructure losses; this is unlikely to be a causal relationship). Notably, including the correlation estimates had little impact on the model results. Median output values remained relatively consistent across all distributions; NPVs increased by 2% for the pessimistic ($-55.25 million) and 12% for the conservative ($-17.52 million) distributions, but decreased by 2% for both skewed distributions (positively biased decreased to $1,109 million, and optimistic decreased to $1,379 million). The standard deviations increased, however, suggesting wider distributions. These results are illustrated in Fig 5 and suggest that correlation among the cost components somewhat increases the likelihood of higher and lower outcomes. These results are relatively consistent across all impact assumptions.

**Table 7:** Correlations between datasets used within the sensitivity analysis^a^.

Correlations	Suppression Variable	Suppression Fixed^b^	Property, Asset and Infrastructure Losses	Timber Losses	Evacuation Costs	Health Costs
Suppression Variable	1.00					
Suppression Fixed	0.68	1.00				
Property, Asset and Infrastructure Losses	0.47	0.00	1.00			
Timber Losses	0.79	0.00	0.42	1.00		
Evacuation Costs	0.34	0.00	0.63	0.57	1.00	
Health Costs	0.77	0.00	0.80	0.60	0.83	1.00

^a^The correlations between datasets are based on a Spearman pair-wise approach within @Risk; values drawn from the dataset distributions are ranked and matched with ranked values from a correlated dataset, defined by the specified correlation. These matched pairs feed into the calculation of correlated model outputs.

^b^Based on the definition of fixed vs variable suppression costs (Table 1), the model assumes correlation coefficients of zero between fixed suppression costs and most cost components, excluding variable suppression costs. We assume a causal relationship between fixed and variable suppression costs (i.e., rising variable costs may lead to an increase in fixed spending), but assume fixed suppression costs do not impact any other cost component. All other correlations are assumed to be a result of changes in variable suppression costs, or adjustments to fire activity.

## Concluding comments

WildFireSat, a satellite mission designed to support wildfire management in Canada, is intended to assist provincial, territorial, and federal agencies make more informed wildfire management decisions, possibly decreasing suppression costs and other negative wildfire-related impacts. We compared direct satellite mission costs to several concomitant or anticipated benefits (avoided losses) of WildFireSat, in a counter factual simulation model augmented with Monte Carlo simulation. Pre-launch, it is impossible to know exactly how WildFireSat will affect wildfire management, hence we developed a set of assumptions representing various expert opinions about the direction and magnitude of impact WildFireSat decision-support products may have. We challenged these assumptions via an extensive sensitivity analysis. Our model has been designed to assess the veracity of these assumptions, leaving room for examining a wide range of possible net results. This is critical given the stochastic and uncertain conditions faced by wildfire managers. Our results suggest the possibility of positive and negative outcomes. Depending on the assumptions associated with the anticipated impacts, mission costs could be between 1.16 and 1.59 times greater than the anticipated benefits, although the model suggests that costs are likely to be nearly equal to the benefits accrued under the pessimistic and conservative assumptions. Alternatively, the benefits could be between 8.72 and 10.48 times greater than the costs; benefits in excess of costs are much more likely under the positively biased and optimistic assumptions. Our cost analysis was restricted to those benefits anticipated from changes in wildfire management; although not included here, the benefits accruing to other economic, environmental, and social sectors would increase the benefit calculations associated with WildFireSat. Although we have made the simplifying assumption that WildFireSat will be equally useful in all situations, it must be acknowledged that the impacts of the mission will be dependent on the type and location of the particular fire situation encountered. WildFireSat decision-support data products may have a large impact in some situations (e.g., an extreme day with multiple ignitions, a fire threatening a high value asset, etc.), and a limited impact in others (e.g., wet fire seasons, fire activity beyond the capacity of suppression resources, etc.). Preventing or mitigating one future extreme event may economically justify WildFireSat many times over. Mamuji and Rozdilsky [69] estimate the total costs of the 2016 Horse River wildfire complex that burned the community of Fort McMurray in Alberta at over $9 billion, more than the costs associated with the WildFireSat mission. Unfortunately, forecasting specific future wildfire situations (e.g., extreme season starts, slow season starts, extreme events, etc.) and within-year fire weather conditions are difficult [70] and beyond the scope of our current model. Our analysis also assumes that the 2013–2018 period is a sufficient and reasonable representation of future conditions. Although this decision was largely based on data limitations, the future in which WildFireSat operates may not be adequately represented by the 2013–2018 period. Indeed, the wildfire situation in Canada in 2023 suggests that what was once thought of as extreme may become a more commonplace occurrence under a changing climate [5]. Anticipated shifts in wildfire activity due to climate change may influence the number of extreme fire years and corresponding loss events in the near future, effecting the costs and social impacts associated with wildfire [9,71,72].

Until real applications are experienced and counterfactual outcomes subsequentially examined, these types of ex-ante analyses will always require judgment. Although our own understanding of WildFireSat and the possible impacts of the satellite mission may sway our analysis somewhat, we believe that our methodology addresses this uncertainty and bias through both the Monte Carlo approach and sensitivity analyses. Our ability to solicit opinions beyond a relatively small group of experts was limited. Despite this, their professional opinions are likely representative of wildfire management agencies across Canada. Other provinces and territories have similar objectives, constraints, and decision-making systems, and have similar familiarity with remote sensing data [60].

In addition to these identified methodology issues, the limited variety of benefits addressed within this analysis should also be acknowledged. We excluded possible benefits associated with improvements to fire crew and public safety, environmental and atmospheric insights associated with fire activity, and reductions in wildfire suppression GHG emissions (e.g. reducing the need for airborne surveillance decreases aircraft emissions). Use of WildFireSat-provided data outside the immediate sphere of wildfire management is also feasible, and could provide other economic, environmental, and social benefits [73]. For example, Environment and Climate Change Canada intends to use the remotely sensed data as part of their smoke and air quality monitoring programs [74], while Natural Resources Canada will use the data for quantifying carbon emissions in support of international emissions reporting requirements and air quality advisories, and Health Canada is likely to use WildFireSat data to assist with efforts to map and monitor urban heat islands (G. Richardson, Health Canada, pers.com).

This analysis explores the known costs and possible benefits WildFireSat could have in a Canada. Our approach is necessarily *ex-ante* in nature, given that the satellite system is still in the design and development phases. We employed an innovative approach to estimate the possible benefits of WildFireSat, expanding on similar methodology that attempts to assign a value to novel datasets (Bard [25] and Gould et al. [30]). The methods applied here identified some of the possible impacts of the satellite-derived data products, emphasizing the process by which these data products could bring about change within the wildland fire management community. We made informed assumptions about the direction and magnitude of these changes to quantify them from an economic perspective. Thus this methodology is arguably more nuanced than some of the value of information analyses that are often used to estimate the benefits of satellite-derived Earth observation data [20].

Future work could look at the impacts of WildFireSat could include forecasting changing fire activity as a result of climate change and projecting cost impacts, and expanding the pool of expert elicited opinions to include additional end-users as data products are adopted. Building on our findings, future work could also evaluate the direct benefits of the mission to specific Canadian wildland fire management agencies, and the implications of expanding adoption of WildFireSat data products to a global scale.
